# Supplementary material for: The Clinical Need for New Diagnostics in the Identification and Management of Patients with Suspected Sepsis in UK NHS Hospitals: A Survey of Healthcare Professionals
Source: Antibiotics (Basel). 2020 Oct 26;9(11):737. doi: 10.3390/antibiotics9110737 (PMC7693654; doi:10.3390/antibiotics9110737)
Supplement: Supplementary file 1 [file antibiotics-09-00737-s001.pdf]

The survey
Information sheet and consent form

Dear Participant,

Thank you for reading this information sheet.

The aim of this survey is to understand the clinical need for a test to support the management of sepsis, to support mapping the care pathway for sepsis and understand its variability across UK.

You have been invited to participate to the survey because you are a practising nurse or doctor in UK with experience in managing sepsis.

You do not have to take part in the survey, and you can stop completing the survey at any point without giving a reason. A decision to withdraw at any time, or a decision not to take part, will not affect your legal rights, or your ability to have future involvement with the DEC Newcastle.

The survey should take around 10-12 minutes to complete. No personal or sensitive data is collected, and your response is anonymous and will be kept confidential. No patient data are collected through the survey. The data collected through this survey may be used in conjunction with data obtained by other Trust-sponsored studies. There are no risks associated to taking part to this survey. Results will be disseminated through publications in scientific journals, conferences and relevant websites.

The survey is being run by the NIHR Diagnostic Evidence Co-operative (DEC) Newcastle (which is delivered as a partnership between the Newcastle upon Tyne Hospitals NHS Foundation Trust and Newcastle University). This survey is part of a larger project funded by an Innovate UK Biomedical Catalyst grant to MOLOGIC Ltd for supporting the development of a new test for sepsis. The NHS Health Research Authority (HRA) have approved this survey for NHS R&D (IRAS ID230491). The study was also deemed eligible for adoption onto the NIHR portfolio (CPMS ID **35683**).

If you have any questions about the research or about the instructions at any stage, please do not hesitate to contact me at: sara.graziadio@newcastle.ac.uk (Senior methodologist, DEC Newcastle). If you have any complaints you can contact the DEC Newcastle deputy director Dr Michael Power at Michael.Power@newcastle.ac.uk.

By clicking on the link below you consent to participate to this study

Thank you for taking part,

Sara Graziadio PhD

Before starting the questionnaire, please check if you are eligible:

- You are older than 18 years old
- You are a registered nurse or doctor
- You work in a hospital in the UK
- You have experience in identifying and/or managing patients with suspected sepsis

Definitions and acronyms

In this survey when we refer to 'sepsis' we have in mind the new definitions established by the report 'Third International Consensus Definitions for Sepsis and Septic Shock (Sepsis-3)' (Singer, 2016).

The definitions and other relevant acronyms are given below:

NEWS: The National Early Warning Score allocates scores to physiological measurements of respiratory rate, oxygen saturation, temperature, systolic blood pressure, pulse rate, and level of consciousness. The total score is increased if supplemental oxygen is required.

Red Flags (RCEM summary of NICE guidance)**:**
Responds only to Voice, Pain or Unresponsive
Systolic blood pressure ≤90mmHg
Heart rate >130 bpm
Respiratory rate >25 pm
Needs oxygen to maintain SaO2 >92%
Non-blanching rash/ mottled/ cyanotic
Has not passed urine in the last 18 hours
Urine output <0.5mL/kg/hr
Lactate ≥2mmol/L
Recent chemotherapy

Sepsis: life-threatening organ dysfunction caused by a dysregulated host response to infection. For clinical operationalization, organ dysfunction can be represented by an increase in the Sequential Organ Failure Assessment (SOFA) score of 2 points or more.

Suspected sepsis: (NEWS≥5 OR Red Flag) AND signs and symptoms of infection.

To characterize respondents

Where do you work?

- ☐ England
- ☐ Wales
- ☐ Scotland
- ☐ Northern Ireland

In which trust do you work? [This information will be used to evaluate inter-trust variability versus intra-trust variability. The trust name(s) will be kept anonymous in the dissemination of the results of the survey.]

Drop-down menu

Other *Free text*

In which section(s) of the hospital do you work?
Tick all applicable

- ☐ Emergency department
- ☐ Ward
- ☐ Intensive care unit
- ☐ Assessment suite
- ☐ Other *Free text*

What is your role within the hospital?

- ☐ Nurse
- ☐ Trainee doctor
- ☐ Non-consultant career grade doctor
- ☐ Consultant

What is your primary specialization?

- ☐ Anaesthesia
- ☐ Infectious disease
- ☐ Microbiology
- ☐ Acute medicine
- ☐ Intensive care
- ☐ Oncology
- ☐ Surgery
- ☐ Paediatrics/Neonatology
- ☐ None (e.g. trainee on rotation)
- ☐ Other *Free text*

Which patient groups do you mostly see?

- ☐ newborns
- ☐ children
- ☐ adults

Please refer to this clinical population when you answer the rest of questions of the survey

How many years' experience do you have post-qualification?

- ☐ Less than 1
- ☐ Between 1 and 4
- ☐ Between 5 and 9
- ☐ 10 or more

How many cases of suspected sepsis have you identified/managed in your career?

- ☐ less than 5
- ☐ between 5 and 50
- ☐ more than 50

To investigate useful test characteristics

<<<all>>>
The main aim of this survey is to understand the clinical need of a test to support management of sepsis. Please indicate the clinical value that novel tests would have in the following scenarios (the value is 1 if the test would be useless, and 10 if it would be most useful)

- ☐ Test for pre-hospital identification of patients at high risk for sepsis (i.e. primary care and ambulance). Results of the test would inform decision on referral
- ☐ Test to rule in infection in hospital in patients with NEWS >=5. Results of the test would inform starting or continuing antibiotic treatment
- ☐ Test to rule out infection in hospital in patients with NEWS >=5. Results of the test would inform stopping or postponing antibiotic treatment
- ☐ Test to assess prognosis in hospital in patients with suspected sepsis. Results of the test would inform risk of organ failure and death
- ☐ Test to guide management of patients who are treated for sepsis. Results of the test would guide antibiotics de-escalation or stopping
- ☐ Test to guide management of patients who are treated for sepsis. Results of the test would inform discharge from ICU
- ☐ Test to guide management of patients who are treated for sepsis. Results of the test would inform discharge from hospital

Do you have any comment on the above scenarios or would you like to suggest other relevant scenarios?
Free text

<<<all>>>
To facilitate the identification of patients with suspected sepsis would you prefer a lab-based test or a near to patient test (often called point of care test)?

- ☐ Lab test
- ☐ Near to patient test
- ☐ Unsure

Why? *Free text*

<<<all>>>
Mologic ltd is developing a device that will enable near to patient testing for sepsis in the ward and/or ICU. It uses a small amount of blood from a venous or arterial venepuncture and results will be available in 10 minutes.

Would a test that detects patients with infection and predicts deterioration in 10 minutes be clinically useful?

- ☐ Yes
- ☐ No
- ☐ Unsure

Why? *Free text*

We are developing a high-level care pathway map for patients with suspected sepsis. We aim to understand the variations in practice across different hospitals and regions. In particular, we would like to understand relevant sources of management delays and the potential clinical need for new diagnostic tests.

<<<**all**>>>
What are the common cause for delays in management of patients with suspected sepsis in your Trust? (Tick all that apply)

- ☐ Identification of patients with suspected sepsis
- ☐ Flagging up of patients with suspected sepsis for review by consultant
- ☐ Consultant review
- ☐ Implementation of Consultant's instructions
- ☐ Assessment by a member of the critical care team
- ☐ Flagging up of deterioration of patients being treated for sepsis
- ☐ No major delays in my trust in the management of sepsis
- ☐ No major delays during day, but some delays present during the nights and in the weekends
- ☐ Unsure
- ☐ Others? *Free text*

<<<**all**>>>
What are the main causes of the delays? (Tick all that apply)

- ☐ Shortage of nurses
- ☐ Shortage of junior doctors
- ☐ Shortage of consultants
- ☐ Shortage of laboratory technicians
- ☐ Lack of accurate diagnostic tests
- ☐ Lack of rapid diagnostic tests
- ☐ Lack of specific sepsis identification and management training for nurses
- ☐ Lack of specific sepsis identification and management training for junior doctors
- ☐ Unsure
- ☐ Others? *Free text*

For the following set of questions we are particularly interested in what usually happens in your hospital — not in what should happen.

<<<IF Nurse OR Trainee OR non-consultant>>>
Do nurses in your Trust flag patients with suspected sepsis for review simply because they are deteriorating, or do they flag those patients for review specifically as suspected sepsis?

- ☐ Flagged for deterioration
- ☐ Flagged for suspected sepsis
- ☐ unsure

<<<IF Nurse OR Trainee OR non-consultant>>>
Is it current practice in your hospital to flag a patient for review **for suspected sepsis** if (tick all those that apply)

- ☐ a Red flag alert is identified
- ☐ the NEWS is equal to or higher than 5
- ☐ the NEWS is equal to or higher than 5 AND there is suspicious (or confirmation) of infection
- ☐ there is a Red Flag alert AND there is suspicious (or confirmation) of infection
- ☐ the NEWS is equal to or higher than 5 OR there is a Red Flag alert AND there is suspicious (or confirmation) of infection
- ☐ Unsure
- ☐ None of the above. *Free text*

<<<IF Nurse OR Trainee OR non-consultant >>>
From the moment in which the patient is flagged by the nurse for review, how long does it **usually** take before the patient is reviewed by the clinician who will decide the therapeutic approach for the patient and/or decide their broader management?

- ☐ 0 to 15 minutes
- ☐ 15 to 30 minutes
- ☐ 30 to 60 minutes
- ☐ More than 60 minutes
- ☐ Unsure

<<<IF Trainee OR non-consultant>>>
if a patient is flagged as suspected sepsis, is the blood taken for further analysis and culture **before** antibiotics are administrated?

- ☐ Always
- ☐ Usually
- ☐ Sometimes
- ☐ Rarely
- ☐ Never
- ☐ Unsure

<<<IF Trainee OR non-consultant>>>

From the moment the patient is flagged by the nurse for review, how long does it **usually** take on average before the blood is taken for further analysis and culture?

- ☐ 0 to 15 minutes
- ☐ 15 to 30 minutes
- ☐ 30 to 60 minutes
- ☐ More than 60 minutes
- ☐ Unsure

<IF Nurse OR Trainee OR non-consultant >>>
From the moment the patient is flagged by the nurse for review, how long does it **usually** take before the patient is given antibiotics?

- ☐ 0 to 15 minutes
- ☐ 15 to 30 minutes
- ☐ 30 to 60 minutes
- ☐ More than 60 minutes
- ☐ Unsure

<<< IF Trainee OR non-consultant>>>
Do you have an on-site laboratory for haematology and biochemistry?

- ☐ Yes, for both
- ☐ Yes, for haematology
- ☐ Yes, for biochemistry
- ☐ No
- ☐ Unsure

<<<IF Trainee OR non-consultant>>>
For cases of suspected sepsis, how long does it usually take to have results from the laboratory from the moment the tests are requested by the clinicians?

For lactate levels:

- ☐ less than 5 minutes
- ☐ between 5 minutes and 1 hour
- ☐ more than 1 hour
- ☐ Unsure

For blood tests (haematology and biochemistry):

☐ Less than 1 hour
☐ Around 1 hour
☐ Between 1 and 2 hours
☐ More than 2 hours
☐ Unsure

For culture:

☐ Less than 24 hours
☐ Between 24 than 48 hours
☐ Between 48 than 72 hours
☐ More than 72 hours
☐ Unsure

<<<IF Nurse OR Trainee>>>
Does your hospital have out-reach nurses from ICU who assess ward patients who are critically unwell (including patients with suspected sepsis)?

☐ Yes
☐ No
☐ Unsure

<IF Nurse OR Trainee OR non-consultant>>>
From the moment in which the patient is reviewed by a clinician in the ward, how long does it take before the patient is assessed by a member of the critical care team for potential admission to ICU (if assessment from the critical care team is requested)?

☐ 0 to 15 minutes
☐ 15 to 30 minutes
☐ 30 to 60 minutes
☐ more than 60 minutes
☐ Unsure

<<<IF Microbiology AND non-consultant OR consultant >>>
How frequently is a PCR test for infection performed for patients with suspected sepsis?

- ☐ 100%
- ☐ 76-100%
- ☐ 51-75%
- ☐ 26-50%
- ☐ 1-25%
- ☐ 0%
- ☐ Unsure

<<<IF Microbiology AND non-consultant OR consultant >>>
What proportion of blood cultures requested to assess a potential diagnosis of sepsis are positive?

- ☐ 100%
- ☐ 76-100%
- ☐ 51-75%
- ☐ 26-50%
- ☐ 1-25%
- ☐ 0%
- ☐ Unsure

<<<IF Microbiology AND non-consultant OR consultant >>>
How frequently is MALDI-TOF test performed on positive cultures?

- ☐ 100%
- ☐ 76-100%
- ☐ 51-75%
- ☐ 26-50%
- ☐ 1-25%
- ☐ 0%
- ☐ Unsure

<<<IF trainee OR non-consultant >>>
In what percentage of cases is an antibiotic susceptibility test carried out in patients with suspected sepsis (if blood culture is positive)?

- ☐ 100%
- ☐ 76-100%
- ☐ 51-75%
- ☐ 26-50%
- ☐ 1-25%
- ☐ 0%
- ☐ Unsure

<<<IF trainee OR non-consultant >>>
How frequently is lactate measured in patients with suspected sepsis?

- ☐ 100%
- ☐ 76-100%
- ☐ 51-75%
- ☐ 26-50%
- ☐ 1-25%
- ☐ 0%
- ☐ Unsure

<all>
Do you have any other relevant comments on the current identification and management of patients with suspected or confirmed sepsis?

To investigate laboratory test usage

The following set of questions will help us understand how blood tests are used in clinical practice in diagnosing and managing sepsis.

<<<IF consultant>>>
What blood tests do you use to support the diagnosis of sepsis **in patients with suspected sepsis**? And how often do you use them?

	In 0% of cases	In 1-25% of cases	In 26-50% of cases	In 51-75% of cases	In 76-100% of cases	In 100% of cases
CRP						
PCT						

<<<IF consultant>>>
Do you use any other blood tests to support a diagnosis of sepsis? *Free text*

<<<IF consultant>>>
Are these tests helpful in the diagnosis of sepsis?

- ☐ Yes, very much
- ☐ Yes, somewhat
- ☐ No, not much
- ☐ No, not at all

<<<IF consultant>>>
What blood tests do you use to postpone the administration of antibiotics **in patients with suspected sepsis**? And how often do you use them?

	In 0% of cases	In 1-25% of cases	In 26-50% of cases	In 51-75% of cases	In 76-100% of cases	In 100% of cases
CRP						
PCT						

<<<IF consultant>>>
What blood tests do you use to stop the administration of antibiotics **in patients treated for sepsis**?

	In 0% of cases	In 1-25% of cases	In 26-50% of cases	In 51-75% of cases	In 76-100% of cases	In 100% of cases
CRP						
PCT						

<<<IF consultant>>>
Do you use any other blood tests to support monitoring of patients treated for sepsis? Free text
Are these tests helpful in monitoring these patients?

- ☐ Yes, very much
- ☐ Yes, somewhat
- ☐ No, not much
- ☐ No, not at all

<<<IF consultant>>>
Are there any comments that you would like to add on the use of blood tests for manging sepsis?

Free text

The following set of questions will help us understand the quantity of information used on average to decide when to start and stop antibiotics in patients with suspected sepsis.

<<<IF non-consultant OR consultant>>>
In patients with NEWS $\geq$5 or Red Flag alert, when do you usually start antibiotic therapy? Please select the scenarios that is more common.

☐	When patient shows symptoms and signs consistent with infection
☐	Not until the results from the blood tests (e.g. white cell count and CRP) are consistent with infection
☐	Only when results of positive cultures are available
☐	Only when antibiotic sensitivities are available
☐	Unsure
☐	Other? *Free text*

<<<IF non-consultant OR consultant>>>
In what circumstances do you usually stop antibiotics in patients who are being treated for sepsis? Please select the scenario that is more common.

☐	When the patient improves clinically, i.e. assessed by symptoms and signs irrespective of blood test results
☐	When the patient improves clinically (i.e. assessed by symptoms and signs) consistent with blood test results
☐	When the course of antibiotics has completed (independently of the patient status)
☐	When the course of antibiotics has completed and there is clinical improvement (assessed by

	symptoms and signs)
☐	When the course of antibiotics has completed and there is clinical improvement (assessed by symptoms and signs, and consistent with blood test results)
☐	Unsure
☐	Others? *Free text*

The following set of questions will help us understand the potential role of a novel test to facilitate the management of sepsis.

The scenarios reported below imply a temporal sequence of increasing acquisition of information from bedside assessment to retrospective examination of the patient at discharge. Their aim is to understand the added value to diagnostic accuracy of each additional test result.

<<<IF consultant>>>
When using bedside clinical assessment alone, what percentage of patients can be confidently diagnosed as having sepsis?

- ☐ 100%
- ☐ 76-100%
- ☐ 51-75%
- ☐ 26-50%
- ☐ 1-25%
- ☐ 0%
- ☐ Unsure

<<<IF consultant>>>
When using blood tests in addition to bedside clinical assessment, what percentage of patients can be confidently diagnosed as having sepsis?

- ☐ 100%
- ☐ 76-100%
- ☐ 51-75%
- ☐ 26-50%
- ☐ 1-25%
- ☐ 0%
- ☐ Unsure

<<<IF consultant>>
When using culture results in addition to bedside clinical assessment and blood tests, what percentage of patients can be confidently diagnosed as having sepsis?

- ☐ 100%
- ☐ 76-100%
- ☐ 51-75%
- ☐ 26-50%
- ☐ 1-25%
- ☐ 0%
- ☐ Unsure

<<<IF consultant>>>
What percentage of patients can be confidently diagnosed ***retrospectively*** as having had sepsis when all available test results and the outcome are taken into account (bedside assessments + blood tests+ cultures+ patient outcome)?

- ☐ 100%
- ☐ 76-100%
- ☐ 51-75%
- ☐ 26-50%
- ☐ 1-25%
- ☐ 0%
- ☐ Unsure

<<<IF consultant>>>

There are many reasons for not admitting patients with suspected sepsis to ICUs. Please indicate how frequently the following reasons for not admitting a patient with suspected sepsis to ICU occur.

	In 0% of cases	In 1-25% of cases	In 26-50% of cases	In 51-75% of cases	In 76-100% of	In 100% of cases	Unsure
Lack of beds in ICU							
Patient was not ill enough to need ICU care (e.g. not at sufficiently high risk of organ failure)							
Patient was too ill to benefit from ICU (e.g. in palliative care)							
Patient was too ill to benefit from ICU because of a late diagnosis of sepsis							
Reason for not admitting the patient was not clear							
Others?							

<<<IF consultant>>>
There are some patients with suspected or confirmed sepsis who die despite appropriate and timely management, and a test for early identification of sepsis is unlikely to change their clinical outcome. How common is this scenario in your experience?

- ☐ 100%
- ☐ 76-100%
- ☐ 51-75%
- ☐ 26-50%
- ☐ 1-25%
- ☐ 0%
- ☐ Unsure

<<<IF consultant>>>
Do you have any comment that you would like to add?

Free text

Additional Information to characterize respondents

What is your gender?

- ☐ Female
- ☐ Male
- ☐ Prefer not to say

What type(s) of NHS acute Trust(s) do you work in?
Tick all applicable

- ☐ District general hospital
- ☐ Tertiary / teaching hospital
- ☐ Other? *Free Text*

Are you aware of the recent recommended change in the definition of sepsis?
(The Third International Consensus Definitions for Sepsis and Septic Shock (Sepsis-3), Singer, 2016)

- ☐ Yes
- ☐ No
- ☐ Unsure

Do you have any other comments to add about any of the topics relevant to this survey?

Free text

Thank you very much for taking time to answer these questions.
